# Bleach correction ImageJ plugin for compensating the photobleaching of time-lapse sequences

**DOI:** 10.12688/f1000research.27171.1

**Published:** 2020-12-21

**Authors:** Kota Miura

**Affiliations:** 1 Nikon Imaging Center, University of Heidelberg, Heidelberg, 69120, Germany; 2 Centre for Molecular and Cellular Imaging, EMBL, Heidelberg, 69117, Germany

**Keywords:** Fluorescence microscopy, photobleaching, bleach correction, histogram matching, restoration, time series, Fiji, ImageJ

## Abstract

During the capturing of the time-lapse sequence of fluorescently labeled samples, fluorescence intensity exhibits decays. This phenomenon is known as 'photobleaching' and is a widely known problem in imaging in life sciences. The photobleaching can be attenuated by tuning the imaging set-up, but when such adjustments only partially work, the image sequence can be corrected for the loss of intensity in order to precisely segment the target structure or to quantify true intensity dynamics. We implemented an ImageJ plugin that allows the user to compensate for the photobleaching to estimate the non-bleaching condition with choice of three different algorithms: simple ratio, exponential fitting, and histogram matching methods. The histogram matching method is a novel algorithm for photobleaching correction. This article presents details and characteristics of each algorithm based on application to actual image sequences.

## Introduction

In biological ﬂuorescence microscopy, cells are irradiated with excitation light that causes the emission of ﬂuorescence from protein markers. This irradiation is necessary to detect the position of proteins but it also has a side effect. Emitted ﬂuorescence gradually decreases by time. This is because, with a certain probability, fluorophores irreversibly lose the ability to fluoresce. This effect is called “photobleaching” and is a widely-known problem in bioimage analysis. Photobleaching not only degrades the visual quality of the results but also interferes with the measurement of molecular kinetics and the quality of the segmentation of target objects. Since bleaching attenuates total signal intensity even when the density of labeled protein is unchanged, precise estimation of the amount of protein or the boundary of the biological structure becomes difficult.

To overcome the problem of photobleaching, improvements can be made either before or after the experiment. Before the experiment, one can tune instruments and sample environment, such as careful choice of the fluorophore, use of anti-fading reagents, decreasing the power of laser irradiation, increasing the detector gain, and increasing the time interval of capturing ^1^. After the experiment, time-lapse sequence image data can be processed to compensate for the loss of intensity. To do this, the amount of the fluorescence loss is estimated and then the none-bleached condition is restored by image processing. We call such restoration procedure “bleach correction”. Several different algorithms have been developed and used by various researchers.

A conventional method for correcting the bleaching has been done by multiplying the inverse of the ratio of intensity loss compared to a reference image frame. Estimation of bleach ratio could either be calculated directly ^2– 4^ or by fitting exponential equation ^5, 6^. We call the first method *simple ratio method* and the second method *exponential fitting method*.

The simple ratio method seems to be the most widely used method. It has been applied for improving the deconvolution results in 3D stacks ^7^ and in the fluorescence recovery after the photobleaching (FRAP) technique. In FRAP literature, this method is called double normalization ^2, 3^. In ImageJ, this method was implemented as a macro by Jens Rietdorf and has been available since 2004 ( https://www.embl.de/eamnet/html/bleach_correction.html).

In the exponential fitting method, it also compensates the loss by multiplying the inverse of the bleach ratio, but the method first fits the exponential equation to the bleaching curve and uses the fitted parameters for deriving the bleach ratio at each time point ^8, 9^. This method has been implemented as an ImageJ plugin and is available as a part of MBF ImageJ for Microscopy bundle ( https://imagej.net/MBF_Plugin_Collection). A plugin PixBleach allows three different exponential decay models to be used for the correction ( http://bigwww.epfl.ch/algorithms/pixbleach/) ^10^.

The histogram matching method is based on a strategy that is different from the above two methods and a novel algorithm introduced in this paper. Instead of correcting the fluorescence intensity based on the average intensity of the image at each time point, the histogram matching algorithm ^11^ is used to restore comparable intensity histogram distribution of each frame by taking the first frame as the reference.

We implemented an ImageJ plugin that allows bleach correction with a choice from these three different algorithms. In this article, we explain each of these algorithms in detail and compare their characteristics.

## Methods

### Implementation

In the case of the simple ratio and exponential fitting methods, the mean intensity of 3D stack from each time point was calculated to estimate the bleach ratio. For the histogram matching method, pixel intensity histogram was generated from the 3D stack at each time point.

### Simple ratio method

We consider an *i*-th frame image *I _i_* = *I _i_*( *x*, *y*) in an image sequence and correct its loss of fluorescence emission. We assume that the mean intensity $\bar{I}$ is constant through the time-lapse sequence if not for the photobleaching. Then the ratio of the mean intensity of *i*-th frame ${\bar{I}}_{i}$ to that of the first frame ${\bar{I}}_{0}$ is the ratio of none-photobleached fluorophores in *i*-th frame. We could then estimate the true pixel intensity of the *i*-th frame by the equation below.

$$I_{i}^{c}(x,y)=\frac{{\bar{I}}_{0}-I_{b}}{{\bar{I}}_{i}-I_{b}}(I_{i}(x,y)-I_{b})$$

*I _b_* is the value of the background intensity. This value is estimated independently by measuring the none-fluorescence region within the image, or by measuring a blank image with all the image acquisition conditions being the same but without sample.

### Exponential fitting method

The bleach correction tool included in MBF ImageJ bundle curve-fits total intensity value of each time frame with an exponential decay curve. This decay curve is then used to estimate the bleach ratio at each time point to calculate the true fluorescence intensity ^12^. We implemented a similar capability for processing three-dimensional time series. In this case, the mean intensity of each time point, the average of 3D stack pixel values, was first fitted by an exponential equation to estimate the background intensity.

$${\bar{I}}_{i}^{\prime }\;(x,y)=ae^{-bi}+c$$

Values *a*, *b* and *c* are estimated by this curve fitting. The original image is then subtracted by the estimated background value *c*. The background-subtracted image was then fitted again with the single exponential equation. Using the estimated parameters from this second fitting, which we now call them *a* *'*, *b* *'*, and *c* *'*, the ratio of bleaching was determined and then it’s inverse was multiplied to the background-subtracted image.

$$I_{i}^{c}\;(x,y)=\frac{a^{\prime }+c^{\prime }}{a^{\prime }e^{-b^{\prime }i}+c^{\prime }}(I_{i}(x,y)-c)$$

### Histogram matching method

Histogram matching algorithm modifies pixel values of an image to match its histogram shape to a reference image histogram ^13^. We used the histogram of the first frame image *H*_0_( *p*) as a reference and matched the histogram of *i*-th image frame *H _i_*( *p*). *p* is the pixel value that is 0 ≤ *p* ≤ 255 in 8-bit image and is 0 ≤ *p* ≤ 65535 in 16-bit image. The cumulative distribution function of histogram *CDF _i_*( *p*) is used for the actual calculation.

$$CDF_{i}(p)=\sum_{x=0}^{p}H_{i}(x)$$

Since we take the first frame of the time-lapse sequence as the reference, we use *CDF*_0_( *p*) as the reference CDF. We then match the rest of CDF to the reference *CDF*_0_( *p*) by

$$p^{\prime }=CDF_{0}^{-1}(CDF_{i}(p)),$$

where $CDF_{0}^{-1}$ is the inverse function of *CDF*_0 _and *p* *'* is the pixel value updated after matching a pixel value *p* in the original image.

### Operation

For running this plugin with Fiji, any entry-level laptop or desktop machine is sufficient. If one needs to work on a huge image stack, then one should make sure that the RAM has a capacity twice the size of the file size of the image stack. In case of Mac, Mac OS X 10.4 or higher is required to run Fiji.

## Results

**Figure 1.**
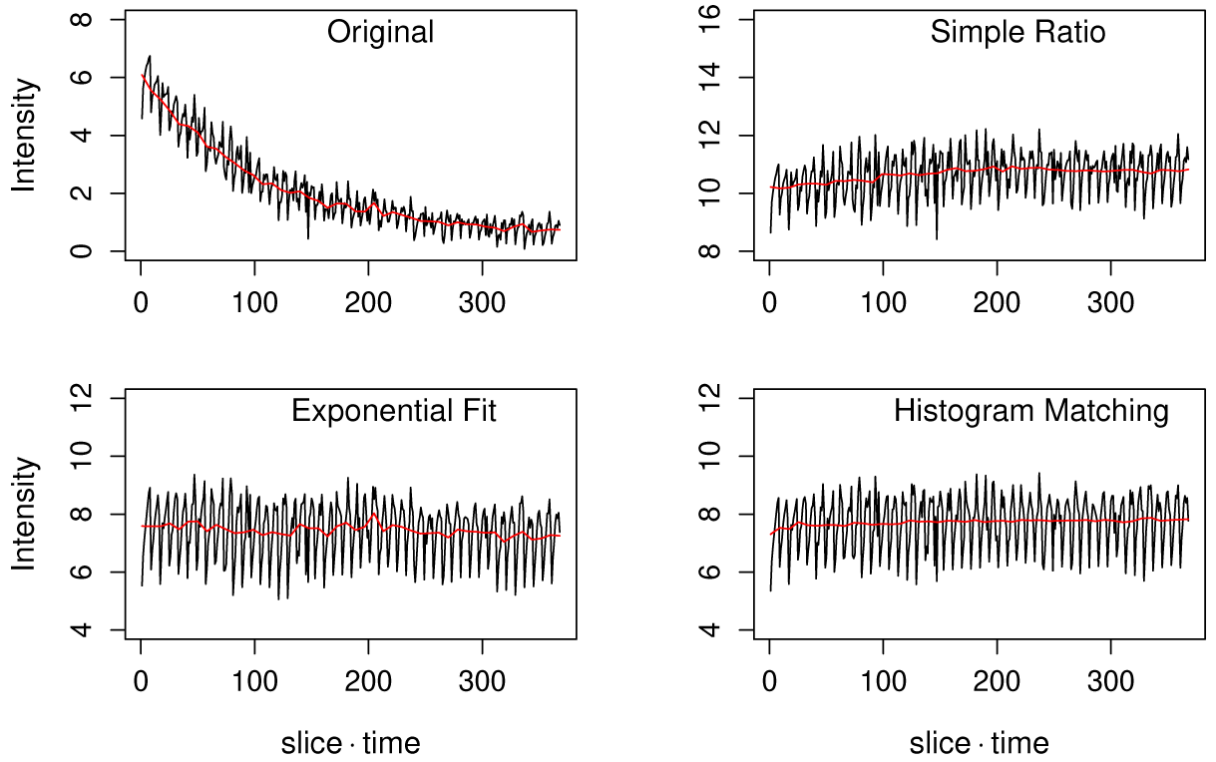
Time course of photobleaching in the original sample sequence (top-left) and corrected curves by the simple ratio method (top-right), the exponential fit method (bottom-left), and the histogram matching method (bottom-right). For the original and the histogram matching results, values were subtracted by 68 to set the y-axis range to a comparable level to the other two curves. Black curves are the mean intensity of each image and red curves are the mean intensity of each time point.

The sample image sequence was a time series of three-dimensional stacks ^14, 15^. The mean intensity showed an overall decrease by time, accompanied by repetitive small peaks ( Figure 1, top-left). These small peaks corresponded to single time points, as each peak represented the spherical shape of the yeast cell. Mean intensity was low at the top slice, high at the cell equator, and then low again at the bottom slice.

### Simple ratio method

To correct for the bleaching using the simple ratio method, we first determined background intensity. An arbitrary area outside the cell was selected and the mean intensity of the full sequence was measured. The mean intensity of the background was 68.3 ± 0.5. The sample sequence was then corrected for bleaching by the simple ratio method using this background intensity ( Figure 1, top-right).

**Figure 2.**
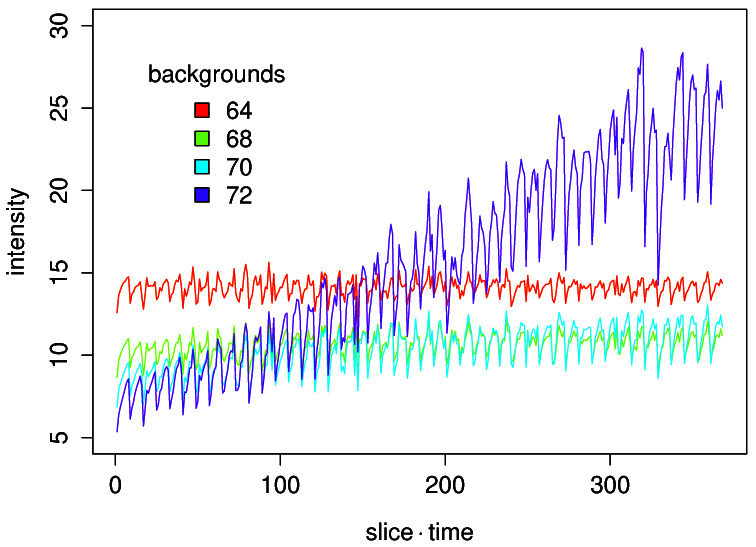
Effect of estimated background intensity on the bleach correction by the simple ratio method.

We further examined how the level of background intensity affects the correction results ( Figure 2). We used four different background values, 64, 68, 70, and 72. A slight difference in the background values caused a large difference in the resulting curves. When the background value was set large, the correction resulted in a curve with an increasing trend. When the value is set small the corrected curve showed a decreasing trend. When the measured background intensity 68 was used, drift was minimal.

### Exponential fitting method

With the exponential fitting method, the estimation of background value is already a part of the algorithm. In the sample image, the estimated background intensity was 73. The image sequence was subtracted by this background value, fitted with a single exponential equation and then the bleaching was corrected ( Figure 1, bottom-left). The mean intensity of the corrected image stack was mostly constant but with a slightly decreasing trend and also with small fluctuations. The decreasing trend was due to a fact that a single exponential equation was not perfectly fitting to the bleaching process seen in the sample. Since the exponential fitting assumes an idealized decrease in fluorescence by time, fluctuations present in the original image sequence were preserved in the corrected image sequence ( Figure 1, red curves).

### Histogram matching method

The histogram matching method does not need background intensity estimation in its algorithm. For the purpose of comparing this method with the other two methods, the original stack was first subtracted by a constant background intensity value 68. The correction resulted in a stably constant mean intensity time series ( Figure 1, bottom-right). It was the most stable result compared to the other two methods in terms of the fluctuation of intensity after the correction ( Figure 1 red curves).

## Discussion

All three methods do correct bleaching of fluorescence but the results of correction showed some difference. The simple ratio method has the capacity to correct time series with abrupt changes in intensity because the bleaching ratio is calculated for each time point. On the other hand, the quality of the correction is heavily dependent on the estimated value of background intensity. A small deviation of estimated value from the true background value causes wrong correction results ( Figure 2).

The exponential fitting method has an assumption that the bleaching process follows a single exponential decay. It is known that in some cases bleaching time course is a double exponential decay ^8^. In this respect, one must consider before using this method if there is any good reason to model the bleaching process of the sample as a single exponential decay. Otherwise, the goodness of fit needs to be evaluated for the proper use of this method. In addition, the exponential fitting method ignores small perturbations in the intensity such as abrupt changes in the emission of fluorescence. Such changes can be caused by small fluctuations in the power of the excitation light or in the slight variations in the timings of the shutter controlling the light path. The simple ratio method deals better with such changes. However, such a non-flexible nature of the exponential fitting method can become an advantage in some other occasions. For example, if the change in the intensity is due to the synthesis of GFP molecules by cell, the simple ratio method will wrongly correct such true increase and mask the biological event, but the exponential fitting method will achieve a better correction as long as the bleaching is known to be a single exponential decay.

The histogram matching method is robust when it is difficult to measure background intensity. This can happen when the whole image frame is filled with sample. For example, image data with packed cells in the image frame hinder the estimation of background intensity. Since histogram matching does not require the input of background intensity, the correction will be straight forward even with such cases. Moreover, this method is especially suited as a preprocessing for segmentation since strictly constant mean intensity in the corrected image sequence affords an optimal condition for segmenting objects.

The limitation of using the histogram matching method is that it assumes a stable distribution of fluorescence. If object under observation undergo changes in localization, *e.g.* signal changes from diffuse to spots during cell surface receptor internalization, or in shape, *e.g.* cell spreading, we expect that histogram shape will change as well. If we take an example of cell surface receptor internalization, the formation of spots by the aggregation of protein is expected to create a new peak in the high pixel values while decreasing the height of the existing peak in low pixel values of the histogram. Applying histogram matching to this time course would result in the wrong correction by forcing the histogram shape to become constant over time.

## Conclusions

All thee methods correct bleaching, but have specific limitations of each. With the simple ratio method, background intensity should be accurately estimated. The exponential fitting method relies heavily on the model. With the histogram fitting method, object shape and pattern should be constant. For choosing an appropriate method, these limitations, and the known details of the observed biological event should be taken into account. In the future, the following features are planned to be added to the plugin: first, a fitting method with a double exponential equation; second, the background estimation method for the simple ratio method will be considered and will be added as a helper function; third, the current histogram matching method uses the first frame as the reference frame. Tolerance to changes in shape and pattern becomes higher if the reference frame is updated for every time frame so that the matching is done between neighboring time points.

## Data availability

### Underlying data

For the development of the plugin, the sample time-lapse sequence of fluorescently labeled yeast cells was kindly provided by Boryana Petrova and Christian Häring (Cell Biology and Biophysics Unit, EMBL Heidelberg). These sequences are three-dimensional time-lapse movies, taken with eight optical sections for each time point ^14^.

Zenodo: A sample image data for ImageJ Bleach Correction Plugin, http://doi.org/10.5281/zenodo.4060111 ^15^.

Data are available under the terms of the Creative Commons Attribution 4.0 International license (CC-BY 4.0).

## Software availability

The Bleach correction plugin is contained within the download package of Fiji ( https://imagej.net/Fiji) and can be used directly by launching the software package and accessing the menu item [Image > Adjust > Bleach Correction] without any further additional installation. For using it in ImageJ 1.× (ImageJ ( https://imagej.nih.gov/ij/), the source code should be downloaded, compiled, and installed locally.

Source code (version 2.0.3) available from: https://github.com/fiji/CorrectBleach

Archived source code as at time of publication: http://doi.org/10.5281/zenodo.58701 ^16^

License: GNU General Public License version 2. 
