# Supplementary material for: Bleach correction ImageJ plugin for compensating the photobleaching of time-lapse sequences
Source: F1000Res. 2020 Dec 21;9:1494. [Version 1] doi: 10.12688/f1000research.27171.1 (PMC7871415; doi:10.12688/f1000research.27171.1)
Supplement: Peer review history

# Peer review: Reviewer response for version 1

**DOI:** 10.5256/f1000research.30015.r77752

**Published:** 2021-02-22

**Authors:** Bram van den Broek

**Affiliations:** 1 BioImaging Faciliy & Division of Cell Biology, The Netherlands Cancer Institute, Amsterdam, The Netherlands

In this paper Kota Miura describes an ImageJ plugin for fluorescence photobleaching correction. This tool has been built into Fiji for a number of years already, and is no doubt used by many researchers. The plugin comprises three different methods to compensate for photobleaching in time-lapse movies (2D/3D). All methods alter the pixel intensities of the output image, in an attempt to stabilize the signal.

 It is praiseworthy that the author takes the effort to describe such an established tool. The article thus also serves as a manual.

 The article is well-written in a very straightforward way, without unnecessary details. The three methods are independently explained to a level that the reader can understand the workflow, but the descriptions don’t go very deep. The discussion is to the point and mainly handles limitations of the implemented methods. The author provides adequate links to software, source code and sample data.

 While reading the paper I had several questions and a few concerns, which I will discuss below. I realize that there are a lot of points; some are very technical and some can be seen as feature requests. I do not expect the author to address/implement them all.

- All example data in the paper is taken from one sample image of a single cell. This image is acquired in 3D, but the bleach correction is performed, or at least shown, in a ‘linearized’ 2D stack. This 2D representation causes periodic ‘peaks’ in the time trace. Perhaps it is the author’s intention to show that the tool works for 3D time-lapses, but these ‘peaks’ obscure the bleaching curve, are unnecessarily confusing the reader and hamper a thorough comparison of the methods. Luckily, the mean intensity of each time point is also provided (the red lines in figure 1).
  Without the black lines, all four panels can be merged into one panel, with different methods in different colors. Doing so will free up room for a few other bleaching correction examples. Ideally, such examples would reflect cases that highlight the positive/negative sides of each method. In my opinion that would really elevate the paper beyond a fancy manual. (I have imaged some data for this review that the author is free to use for this purpose, if desired.).
- The output image in the Simple Ratio method has the background subtracted, where in the other methods the background remains. It would be better to keep this the same for all methods.
  Because the output image is not in 32-bit floating point format, this operation also creates a positive bias in the background noise (negative numbers are set to 0). Even more so, the calculation itself seems to be done in 8-bit or 16-bit integer format. This partially underlies the following issue:
- Figure 2 depicts corrected traces of a single cell surrounded by background, for varying input background estimation, using the Simple Ratio method. The figure seems to indicate that *overestimating* the background is much more severe than *underestimating*. However, the interesting part of the data is obviously the cell, and not the background. The corrected traces are mean intensities of the whole image. A flat trace can arise from the amplified signal of the bleaching cell alone (this is what we want), but also from an erroneously amplified background. With many more background pixels than ‘cell pixels’ this effect can be large even with moderate background increase. Exactly this turns out to be happening with the image stack presented here.
  I have reproduced the bleaching corrected data with the sample image used in the article, but now also separated the foreground (the cell) from background (using a simple Otsu threshold on a slice where the cell is in focus, halfway the time-lapse). The resulting time traces (total, cell, background) show that when the background set at 64, the background intensity *increases* by 45% while the cell intensity *decreases* by 35%: under-correction. With background at 72 the background intensity *decreases* by 42%, while the cell intensity *increases* by 29%: over-correction. (In the latter case the background gray values are almost zero everywhere, because the image remains 8-bit; this could be part of the problem.)
  When a correct background of 68.3 is set (or 69, because non-integer values seem to be rounded up) the cell intensity over time remains roughly constant.
  Because the traces as shown in Figure 2 (and Figure 1 as well!) are computed from the cell+background, which both vary, they do not fully convey the right message.
  Interestingly, the paragraph explaining the effect of background intensity correctly describes the result, but the words do not agree with the figure. Perhaps the author can instead show both cell and background traces (and then preferably only the mean/sum of all z planes vs time, to remove the spikes).
- I noticed that with the Simple Ratio and Exponential Fit methods it is possible to calculate the correction on a selection (ROI) in the image, yielding different results. Please add a paragraph on the effect, and when it is useful to do so.
- Exponential method: The estimated background value *c* is subtracted from the original. Is this done in 32-bit floating point numbers, to prevent a positive bias in the background noise?
- Exponential method: The text says “ *The original image is then subtracted by the estimated background value c. The background-subtracted image was then again with the single exponential equation.*” Why is the data again fitted again after background subtraction? Am I missing something, or would it just lead to *a* = *a*’, *b* = *b*’ and *c*’=0? And if not, why is that?
  When running the plugin the exponential fit is displayed. Are the parameters in this fit plot *a, b,* *c*... or *a*’, *b*’, *c*'?
- Exponential method: The author correctly states that bleaching sometimes (more often than not?) follows a double exponential curve, and that “the goodness of fit needs to be evaluated for the proper use of this method.” Unfortunately, the displayed R ^2^ in the plot is not the same as goodness of fit, and is really not valid for a nonlinear regression. (See for instance Spiess and Neumeyer (2010 ^1^). A real goodness of fit, like Chi ^2^, involves knowing the uncertainty of the signal. I am not asking for that, but for judging the goodness of fit by eye it would be instructive to also show a residuals plot.
- Histogram Matching method: This method yields the best results in some cases, as described, but I also observe strange incorrect behavior on some of my (confocal) bleaching time-lapses (with no change in fluorescence localization, background almost zero): sudden steps, not a stable signal. I’m not sure what to make of that, but I suspect that it involves handling of the background, possibly because of integer rounding. Also with this method, I sometimes see the background increase (in steps), though the effect does not seem as severe as choosing a wrong background in the Simple Ratio case.
- The Histogram Matching method is blazingly fast for 8-bit images, but extremely slow for 16-bit images. The difference at least 1000-fold.
- The output images are all named DUP_[image name]. Renaming them would be nice, e.g. [image name]_BleachCorrected_[method].

Textual changes:

- Abstract: *We implemented an ImageJ plugin that allows the user to compensate for the photobleaching to estimate the non-bleaching condition with choice of three different algorithms – *This **sentence seems incorrect. Replace second to by and?
- Introduction: *A conventional method for correcting the bleaching has been done by multiplying the inverse of the ratio of intensity loss compared to a reference image frame* – sentence not quite correct. Multiplying by the inverse of the relative intensity loss compared to a reference frame?
- *In the exponential fitting method, it also compensates the loss by multiplying* by/with *the inverse of the bleach ratio, but…*
- Methods: *In the case of the simple ratio and exponential fitting methods, the mean intensity of the 3D stack from each time point was calculated to estimate the bleach ratio. For the histogram matching method, the pixel intensity histogram was generated from the 3D stack at each time point.*
- *The histogram matching algorithm modifies pixel intensities…*
- Conclusions: *All th ree methods...*

Are the conclusions about the tool and its performance adequately supported by the findings presented in the article?

Partly

Is the rationale for developing the new software tool clearly explained?

Yes

Is the description of the software tool technically sound?

Yes

Are sufficient details of the code, methods and analysis (if applicable) provided to allow replication of the software development and its use by others?

Yes

Is sufficient information provided to allow interpretation of the expected output datasets and any results generated using the tool?

Partly

Reviewer Expertise:

BioImage Analysis, Microscopy, Biophysics

I confirm that I have read this submission and believe that I have an appropriate level of expertise to confirm that it is of an acceptable scientific standard, however I have significant reservations, as outlined above.

## References

[1] : An evaluation of R2 as an inadequate measure for nonlinear models in pharmacological and biochemical research: a Monte Carlo approach. *BMC Pharmacol*.2010;10: 10.1186/1471-2210-10-6 6 10.1186/1471-2210-10-6 20529254PMC2892436

# Peer review: Reviewer response for version 1

**DOI:** 10.5256/f1000research.30015.r78013

**Published:** 2021-02-16

**Authors:** Anna H. Klemm

**Affiliations:** 1 Division of Visual Information and Interaction, Department of Information Technology and Science for Life Laboratory, Uppsala University, Uppsala, Sweden

The article describes the Fiji plugin Bleach correction, which integrates three methods for correcting intensity loss by bleaching. One of the methods, histogram matching, is introduced and novel for addressing bleaching. All methods are well explained. Methods are compared to each other not only by performance on the given use case but also with other use scenarios in mind. I especially liked that specific biological examples were given (e.g. synthesis of GFP) and how the methods would compare in these scenarios.

 It was no problem to test the plugin with the sample data linked in the text and the description.

**Structure of the article**. Methods and results could be fused, such that the explanation of a method goes directly along with presentation of the correction result on the sample data set. It should be referred to Fig 1 earlier in the text, e.g. Fig 1 top left to illustrate bleaching when introducing it in the very beginning. The chosen structure could be due to guidelines of the publisher and in this case it should be communicated to the publisher.

 Other minor suggestions:

**Introduction**:

- Include a sentence about ImageJ.

**Methods:**

- **Simple ratio method:**
*We could then estimate the true pixel intensity **I _i_^c^** of ..*
  Include the information that the measurement is done per slice.
- **Histogram matching method:**
  Since the article has also educational character it could be good to include example cumulative histograms of a frame before and after correction using the histogram matching method.
*We used the histogram of the first frame* ** ***(per slice?)*** *image H0 (p) as a* *reference..*

**Results**:

- *The sample image sequence was a time series of three-dimensional stacks **of a yeast cell**.*
- **Simple ratio method:**
  Add a brief definition of background to the introduction, and that, due to the different nature of source, it has a different bleaching behavior.
  I liked that the effect of the background intensity value was given much space in the article, including a figure.
- **Exponential fitting method:**
*In the sample image, the estimated background intensity was 73.* *: In the sample image, the background intensity* *estimated by fitting was 73* **(to stress that estimation by fitting is different from the estimation by manually selecting a background region and measuring the intensity within the selection).
  You might want to show the fit together with the curve (as done in the plugin output).
- **Figure 1:**
  Specify that it is a stack imaged over time also in the figure legend.
  Instead of a red line use red dots at the center slice to reflect that it is the average value per time point. Dots in the center slice position can also help to visually check whether the z-position was stable over the time of recording.

Are the conclusions about the tool and its performance adequately supported by the findings presented in the article?

Yes

Is the rationale for developing the new software tool clearly explained?

Yes

Is the description of the software tool technically sound?

Yes

Are sufficient details of the code, methods and analysis (if applicable) provided to allow replication of the software development and its use by others?

Yes

Is sufficient information provided to allow interpretation of the expected output datasets and any results generated using the tool?

Yes

Reviewer Expertise:

BioImage Analysis

I confirm that I have read this submission and believe that I have an appropriate level of expertise to confirm that it is of an acceptable scientific standard.

# Peer review: Reviewer response for version 1

**DOI:** 10.5256/f1000research.30015.r78014

**Published:** 2021-02-09

**Authors:** Thomas Pengo

**Affiliations:** 1 University of Minnesota Informatics Institute, University of Minnesota, Minneapolis, MN, USA

The author has addressed a very common issue in fluorescence microscopy experiments and has provided an easy to use tool that is integrated directly into Fiji. The manuscript is easy to read and complete.

 I particularly appreciate the effort to provide precise references to the software versions.

 It would be nice to have an image illustrating the effects of photo-bleaching and the corresponding compensation algorithms. The author provides plots, but no images.

 I have a few minor comments. The page numbers refer to the PDF version, as appear in the first version 1 of the manuscript.

 Page 3, col.1 

- “before or after the experiment”. Unclear, maybe add “imaging experiment”, or simply “imaging” or “image acquisition” or “image data acquisition”. The experiment, without context, is typically larger in scope and includes, for example, sample preparation.
- “fluorescence loss is estimated and then the none[sic]-bleached condition is restored” is a bit too optimistic. Apart from the typo (non-bleached), I would argue something like “fluorescence loss is estimated and compensated for through image processing” would convey a more accurate assessment.

Page 3, col.2 

- “none-photobleached” => “non-photobleached”
- “none-fluorescence” => “non-fluorescing”? or, better, “an empty region”

Page 4, Figure 1. 

 The plot was confusing at first glance as it mixes two dimensions in one. I’d suggest using either a 3D surface (t, z vs Intensity) or an average/max projection in z  (t, Intensity mean over z). The latter is probably sufficient even though it hides the Intensity variation in z. 

 Page 4, col. 1

 There is no mention of the number of bins in the image. This is relevant for the following reason.

  CDF _0_ can be inverted only if strictly monotonic (CDF _0_(a) < CDF _0_(b) i.i.f. a0 ^-1^ will have two possible values c-1 and c. An easy fix would be to add a small constant to H everywhere (e.g. 1) after calculation.

Are the conclusions about the tool and its performance adequately supported by the findings presented in the article?

Yes

Is the rationale for developing the new software tool clearly explained?

Yes

Is the description of the software tool technically sound?

Yes

Are sufficient details of the code, methods and analysis (if applicable) provided to allow replication of the software development and its use by others?

Yes

Is sufficient information provided to allow interpretation of the expected output datasets and any results generated using the tool?

Yes

Reviewer Expertise:

Image processing and analysis

I confirm that I have read this submission and believe that I have an appropriate level of expertise to confirm that it is of an acceptable scientific standard.

# Peer review: Reviewer response for version 1

**DOI:** 10.5256/f1000research.30015.r78015

**Published:** 2021-02-08

**Authors:** Arianne Bercowsky Rama

**Affiliations:** 1 EPFL (École polytechnique fédérale de Lausanne), Lausanne, Switzerland

The article presents three different methods for the correction of photo-bleaching as a post-processing step using an ImageJ/Fiji plugin. The mathematical details of how the corrections are done in 3-dimensional time-lapse data are explained. Moreover, an example data-set is provided to show the photo-bleach correction results when implementing each method. Limitations and examples for the three methods are described, guiding the user when to apply them. The three methods are: simple-ratio, exponential fitting and histogram matching. This last one is a novel algorithm introduced in this article.

 The software tool described in this article by Kota Miura provides the information needed to understand how the Bleach correction ImageJ/Fiji plugin works. The article is easy to read and offers sufficient details to comprehend the stated methods and its applicability. A few questions were raised while reading the methods section:

- **Exponential fitting method:** Once the background subtracted image is fitted a second time with the single exponential equation, you obtain the values a’, b’ and c’. It is not clear why it is then used the estimated value 'c’ since it seems it should be very close to 0.

- **Operation: **the author suggests that the RAM should have a capacity twice the size of the file size of the image stack. Is it because the new corrected image will be duplicated? For very large data-sets, can Virtual Stack be used?

- **Simple ratio method and exponential fitting method:** In these two methods the background plays an important role in the correction. What happens when the background is non-uniform? Is there a need of a prior step to correct for this in order to use these photo bleach correction methods?

Are the conclusions about the tool and its performance adequately supported by the findings presented in the article?

Yes

Is the rationale for developing the new software tool clearly explained?

Yes

Is the description of the software tool technically sound?

Yes

Are sufficient details of the code, methods and analysis (if applicable) provided to allow replication of the software development and its use by others?

Yes

Is sufficient information provided to allow interpretation of the expected output datasets and any results generated using the tool?

Yes

Reviewer Expertise:

Light Sheet Microscopy, Bio-image analysis

I confirm that I have read this submission and believe that I have an appropriate level of expertise to confirm that it is of an acceptable scientific standard.

# Peer review: Reviewer response for version 1

**DOI:** 10.5256/f1000research.30015.r76514

**Published:** 2021-01-18

**Authors:** Fabrice P. Cordelières

**Affiliations:** 1 CNRS, INSERM, Bordeaux Imaging Center, BIC, UMS 3420, US 4, F-33000, University of Bordeaux, Bordeaux, France

This paper presents a software tool by Kota Miura aimed at correcting image datasets for the effect of photobleaching. Shaped as an ImageJ/Fiji plugin, it provides three different methods: the first is based on a simple ratio, the second on exponential fitting, the third on histogram matching. The latter method is novel to the field. The author describes both the theoretical approaches on which the tools has been built on, gives characterization details and finally concludes on the application field of each method.

 Although the manuscript meets the expectations about what a software paper should be, minor suggestions and questions have been raised when reading the paper.

**Improving the reader’s experience:**

- In spite of the full dataset being available through Zenodo, no image is actually presented within the manuscript. A selection of the original and processed images could be presented alongside the graphs, for visual inspection.
- The conclusion section clearly states the different fields of application/restriction use for each single method. A visual transcript, as a decision tree might help the end-user decide which method to try in her/his first attempts.

**About the methods section:**

- Simple ratio: the author makes the reader aware of the caution to be applied when estimating the background, and its impact on the correction when overestimated. It remains unclear to me how the plugin gets this background estimate: is it automatically estimated (if so, how?) or should the user place a region of interest over the image?
- Exponential fitting: Would it be possible to use background estimate from the exponential fitting method as a starting point to evaluate which value to set when using the simple ratio method? The background estimate is higher than the one used for simple ratio method, but it could be of interest to investigate if a value could be automatically derived from this estimate, if possible/relevant.

**About the discussion section:**

- The author discusses the effect of physiological fluctuation of intensity on the efficiency of the correction algorithms, pointing out the simple ratio method as the most appropriate in such situations. Would pre-processing the data with smoothing algorithms such as applying a sliding average algorithm (with proper window size) help?

Are the conclusions about the tool and its performance adequately supported by the findings presented in the article?

Yes

Is the rationale for developing the new software tool clearly explained?

Yes

Is the description of the software tool technically sound?

Yes

Are sufficient details of the code, methods and analysis (if applicable) provided to allow replication of the software development and its use by others?

Yes

Is sufficient information provided to allow interpretation of the expected output datasets and any results generated using the tool?

Yes

Reviewer Expertise:

Bio-image analysis

I confirm that I have read this submission and believe that I have an appropriate level of expertise to confirm that it is of an acceptable scientific standard.
